# Clearance of oxytocin and its potentially enzyme resistant analogues in the *OXT*‐receptor compartment of the potassium depolarized rat myometrium

**DOI:** 10.1002/psc.3372

**Published:** 2021-10-14

**Authors:** Vladimir Pliska, Guido Jutz

**Affiliations:** ^1^ Department of Biology Swiss Federal Institute of Technology (ETH) Zürich Switzerland; ^2^ Department of Molecular Biology and Biophysics Swiss Federal Institute of Technology Zürich Switzerland

**Keywords:** carba‐oxytocin, deaminooxytocin, depolarized uterus, rat, oxytocin, enzymatic splitting, oxytocin, ex vivo pharmacokinetics, oxytocin, Free–Wilson analysis, receptor compartment, response kinetics, time–response relationships

## Abstract

The time–response behaviour of a group of oxytocin analogues structurally modified on potential sites of oxytocin splitting by tissue inactivation enzymes (“enzyme probes”) was investigated ex vivo on the potassium depolarized rat myometrium (at 30°C) and compared with the data obtained in the in vivo experiments. The modified oil‐immersion method by Kalsner and Nickerson was used to record time profiles after cessation of a steady state myometrium contraction triggered by analogues in a high potassium tissue medium. An exchange of the aqueous medium for mineral oil enables to suppress return diffusion of the peptide and to record its irreversible clearance near the corresponding receptor compartment. Response records were analysed by a nonlinear numeric procedure based on combination of steady state and kinetic terms that allows concomitant estimations of affinities from time–response measurements, in the given case for analogues on depolarized myometrium. Potential inactivation‐sensitive sites in the oxytocin chain are the *Ν*‐terminal peptide bond Cys^1^‐Tyr^2^ (aminopeptidase splitting), the intramolecular disulphide bridge (reduction and formation of the practically inactive linear peptide) and the *C‐*terminal Leu^8^‐GlyNH_2_^9^ or the Pro^7^‐Leu^8^ (postprolin cleaving enzyme) bond, respectively. Clearance rate constants of single peptides in the OXT‐receptor compartment were in an interval of 0.025 to 0.28 min^−1^. The fragment contribution analysis reveals a significant linear additivity of individual structural changes and thus a predictivity of irreversible inactivation rate in the receptor compartment. The most potent inactivation of oxytocin is associated with aminopeptidase splitting; other enzymes may play some though nondecisive role. Less significant differences within the peptide group were found for rate constants for peptide transport between receptor compartment and its external aqueous medium. Besides rate constants, the evaluation of time–response data yields affinity values of the tested peptides and indicates a 25‐times desensitation of depolarized compared with a native state.

## Graphical abstract

Oil‐immersion method (Kalsner and Nickerson, 1968) has been adapted for ex vivo estimation of the oxytocin displacement rate from its receptor compartment in rat myometrium. “Enzyme probes,” oxytocin analogues protected against enzymatic attack on putative splitting sites, were used to identify irreversible clearance processes of oxytocin. Segment contribution analysis (Free & Wilson, 1964) of corresponding rate constants indicates *N*‐terminal bond splitting as the major inactivation step; reduction of the ‐S‐S‐ bridge or *C*‐terminal splitting are less significant.

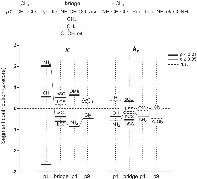

## 1 INTRODUCTION

Neurohypophyseal hormones—oxytocin and vasopressin—are rather short‐acting peptides in current pharmacological in vivo experiments. Their half‐life in the blood plasma lies by human and animal species so far investigated between 1.5 and 8 min.^1^ Regardless of the gender and the reproductive circle by female animals, the half‐life of oxytocin was estimated in rats to 1.65 min^2^; the related elimination rate constant is 0.6 min^−1^. However, estimates of the so‐called overall decay rate constants (*k*_*ρ*_)^3^ for its uterotonic and antidiuretic responses are considerably lower, 0.18 to 0.25 min^−1^. In general, low *k*_*ρ*_ values were reported in a number of reviews also for other neurohypophyseal peptide analogues^3^, ^4^, ^5^, ^6^, ^7^; those for uterotonic response to peptides used in this communication are outlined in Table 1.

**TABLE 1:** Peptides used as enzyme probes: Uterotonic activities and decay rates in vivo (rat)

Symbol	Substance^a^	Common name	Uterotonic activity (rat uterus)^b^		Response decay rate *k*_*ρ*_ (min^−1^)^c^
			In vitro (IU)	In situ (IU)	
OXT	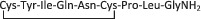	Oxytocin	450 486 507 546 ***490***	468	0.250 0.237 *0.173 ± 0.023*
DOT	[1‐β‐mercaptopropionic acid]‐OXT	Deaminooxytocin	368 551 803 ***677***	476	0.148
HOT	[1‐(2‐Hydroxy‐3‐β‐mercaptopropanoic acid)]‐OXT	Hydroxyoxytocin	1607 1641 ***1624***	*874*	
C_1_OT	[6,1‐Cystathionine]‐OXT	Carba^1^‐oxytocin	368 743 ***743***	120^d^ ***973***	0.277
DC_1_OT	[1,6‐(2‐Amino‐4‐thiasuberic acid)]‐OXT	Deamino‐carba^1^‐oxytocin	1899	1206 1251 ***1229***	0.172
C_1,6_OT	[1,6‐α′,α‐diaminosuberic acid]‐OXT	Dicarba‐oxytocin Carba^1,6^‐oxytocin	5.4		
DC_1,6_OT^e^	[6,1‐(α‐aminosuberic acid)]‐OXT	Deamino‐dicarba‐oxytocin	93	95	0.079
DC_6_OT	[6,1‐(2‐Amino‐4‐thiasuberic acid)]‐OXT	Deamino‐carba^6^‐oxytocin	929	2792 ***746***	0.041? 0.127 (interpolated)
AOT	[9‐Azaglycine]‐OXT	Azaglycine‐oxytocin	≈700		
DAOT	[1‐β‐mercaptopropionic acid, 9‐azaglycine]‐OXT	Deamino‐azagylcine‐oxytocin	1099		
GOT	[4‐Glutamic acid‐δ‐methylester]‐OXT		10.2^f^ 18^g^		
DGOT	[1‐β‐mercaptopropionic acid, 4‐glutamic acid‐δ‐methylester]‐OXT		21.4^f^ 43^g^		

^a^ Synthesis, properties and detailed nomenclature are summarized in K. Jošt, M. Lebl and F. Brtník (eds): *CRC Handbook of Neurohypophyseal Hormone Analogs,* vol. II, CRC Press, Inc., Bota Racon, FL, 1987 (pp. 127–267).

^b^ Uterotonic activities international units defined by *The Third International Standard for Oxytocic, Vasopressor, and Antidiuretic Substances* ^8^ in international units (IU) per mg peptide. Data by various authors are shown in upper rows, adjusted values (see Section 3) in fat italics. In vitro activities relate to estimates in Mg^2+^‐free tissue medium. Oxytocin (adjusted value) was used as a local reference peptide.

^c^ Rate constant of response decay (earlier formal elimination constant).^9^ Data: Barth et al.,^4^ Pliška^3^ (in italics), values denoted by a question mark (?) are outliers (corresponding values corrected by interpolation are specified; see text).

^d^ Value quoted in the thesis of O. Keller (Diss. ETH 5325, 1974) considered as a preliminary estimate. Adjusted value was attained by interpolation (see Result section).

^e^ Not used in the oil‐immersion experiments presented here.

^f^ Pliška and Rudinger^10^

^g^ Photaki et al.^11^

The time span of a response to neurohypophyseal peptides became an important factor in the clinical pharmacology. Structural changes potentially resulting in its prolongation were already subjects of early studies,^12^ and several prolonged analogues found their place in medicine. Thus, long‐acting 1‐deamino‐8‐D‐arginine‐vasopressin (dDAVP, Desmopressin INN) is currently utilized as the preferential drug in the substitution therapy of the central form of *diabetes insipidus* ^13^ and/or as a haemostatic in mild forms of haemophilia A^14^; 1‐triglycyl‐8‐lysin‐vasopressin (Terlipressin INN) is occasionally used as a vasoconstrictor drug in various forms of cardiovacsular collapse and shock.^15^ Carbetocin (INN), 1‐deamino‐2‐O‐methyltyrosin‐carba^1^‐oxytocin,^16^ a long‐acting partial agonist of oxytocin, found a prominent place in emergency obstetrics as a uterotonic and haemostatic drug in critical states of postnatal hemorrhagy^17^ or after caesarian sections.^18^

Earlier results of the pharmacokinetic analysis^19^, ^20^ point out that the response dynamics is predominantly controlled by the drug concentration in the immediate vicinity of the corresponding receptor sites, the so‐called receptor compartment.^12^, ^21^, ^22^, ^23^, ^24^ Thus, kinetics of clearance and drug transport processes are limiting factors in the response duration.

In order to investigate them more directly and to segregate the irreversible clearance from the peptide transport processes, we used a combination of the ex vivo* washout experiments and the oil‐immersion method (assigned here as a “stopped‐transport” procedure), introduced by Kalsner and Nickerson.^25^, ^26^ In our modification,^10^, ^22^, ^27^ it consists in eliciting a steady‐state tonic contraction of an isotonically or isometrically suspended muscle strip ex vivo by a uterotonic stimulant in an aqueous medium, and successively exchanging this medium for a mineral oil. The extreme hydrophobic barrier around the tissue prevents the reverse diffusion of the stimulating agents from the receptor compartment and hence allows following solely irreversible clearance in the response relaxation phase.

**FIGURE 1:**
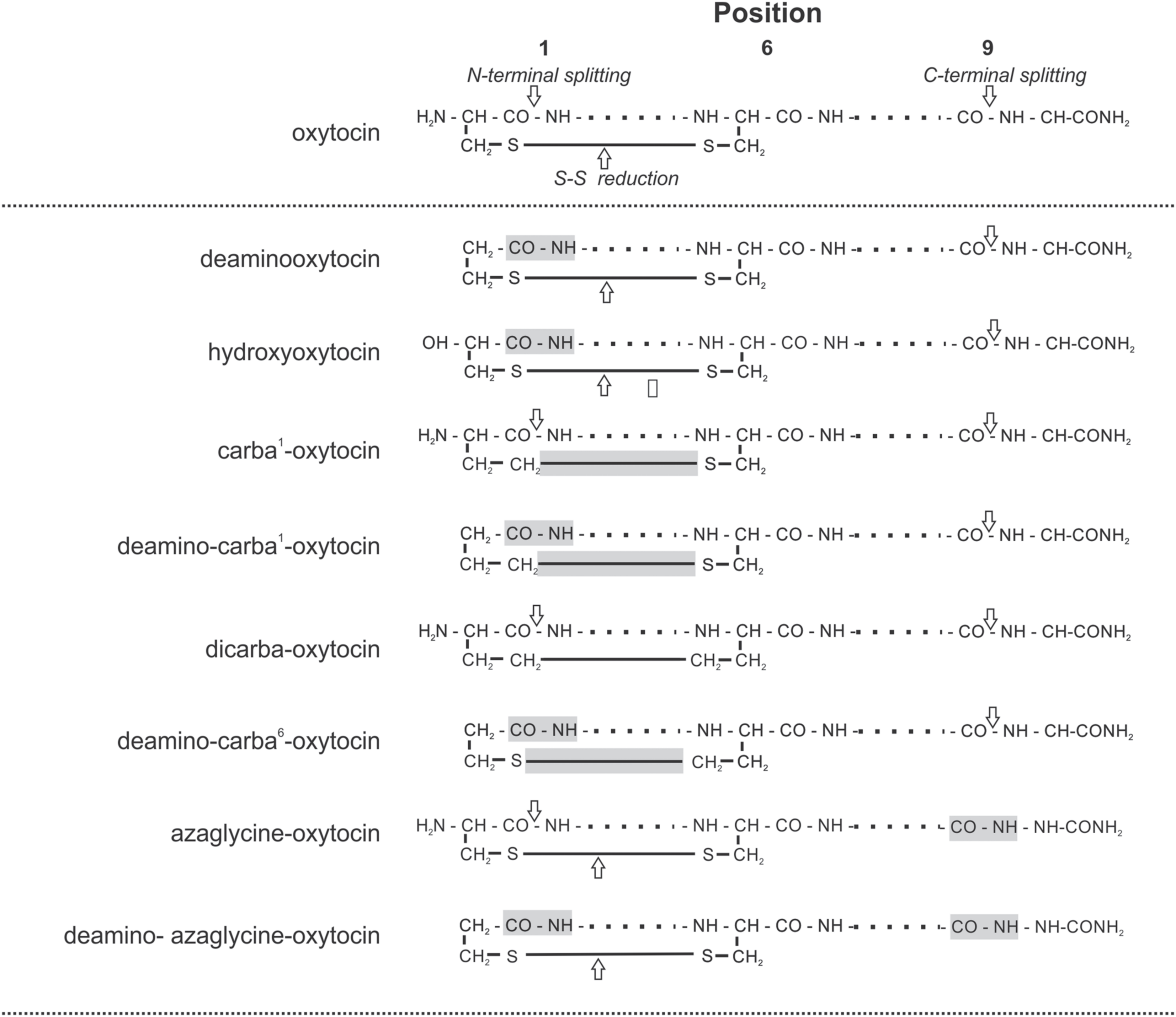
Oxytocin analogues used as enzyme probes: schematic sequences. Arrows indicate the site of potential splitting at sequence sites 1, 6, 9 and at intramolecular ‐S‐S‐ bond, grey areas mark protected sites

The peptide chain of oxytocin carries several potential sites exposed to enzymatic attacks reported in a number of reviews,^28^, ^29^, ^30^, ^31^ and outlined in Figure 1. Splitting the *Ν*‐terminal peptide bond without opening the ‐S‐S‐ bridge by the so‐called oxytocinase,^32^, ^33^ a placental leucine aminopeptidase of the P‐LAP family, was initially detected in the blood serum during pregnancy. Action of other aminopeptidases after enzymatic or nonenzymatic (thiol‐disulphide interchange) reduction of the disulphide bond appears as a likely inactivation step but has not been proved experimentally. The *C‐*terminal hydrolysis of the Leu^8^‐GlyNH_2_^9^ bond by a carboxypeptidase‐type enzyme,^34^, ^35^, ^36^ and the Pro^7^‐Leu^8^ bond splitting^37^ were identified in homogenates of rat and human uteri.

The so‐called “enzyme probes,”^10^, ^12^, ^38^ analogues with potentially enhanced enzyme resistance of individual peptide chain sites, still possessing biological activity, were used to detect sites of oxytocin molecule critical for its clearance in the myometrial oxytocin receptor compartment (OXTR).† Table 1 summarizes those currently employed in our oil‐immersion experiments. Since most of the investigated peptides carry a combination of several protective modifications, the molecular segment contributions analysis according to Free and Wilson^39^ was applied to estimate site contributions of individual structural features to clearance and transport rate constants.

## 2 MATERIALS AND METHODS

### 2.1 Substances

The peptides used here as enzyme probes and their abbreviations are listed in Table 1.

1. Analogues modified on the *N*‐terminal by removing, or altering, the amino group of 1‐cystein are expected to assess sensitivity against aminopeptidase splitting.
2. Substitution of one or both sulphur atoms in the ‐S‐S‐ bridge by a CH_2_ group,^40^ the so‐called carba‐analogues, may clarify the potential clearance role of disulphide reduction.
3. Analogues with a Leu^8^‐Gly^9^ peptide bond (see Figure 1) modified by insertion of aza‐glycine in position 9^41^ were introduced as probes of potential *C*‐terminal cleavage.
4. Furthermore, two analogues esterified in position 4, [4‐glutamic acid‐*δ*‐methylester]‐oxytocin and its *Ν*‐deaminated counterpart (GOT and DGOT, see Table 1), were included in our investigations as a proof of concept^5^, ^10^ as analogues eliciting short‐lasting responses.

Oxytocin was donated by FERRING AB, Malmö, Sweden. The commercial products were purified by counter‐current distribution and stored in lyophilized form. HOT^42^ was supplied by Dr. D. Hope, Oxford, England; AOT^41^ by Dr. H. Niedrich, Berlin‐Friedrichsfelde, Germany; DC_1_OT and DC_6_OT by Drs. K. Jošt and T. Barth, Praha, Czech Republic; GOT and DGOT^11^ by Dr. I. Photaki, Athens, Greece. Deaminooxytocin and DAOT were prepared by Dr. M. Mühlemann, C_l_OT and C_l,6_OT by Dr. O. Keller at the Department of Molecular Biology and Biophysics, ETH Zürich. Uterotonic activities of these analogues were assayed ex vivo on an isolated isometric rat uterus taken from females in natural oestrus, in the bath medium according to Munsick.^43^, ^44^ Activities in IU‡ per mg peptide were collected from literature sources (standard: 3rd International Standard for Oxytocic, Vasopressor and Antidiuretic Substances^8^). Activities assessed in our laboratory (Table 1) were evaluated from dose–response curves according to the scheme suitable for nonparallel peptide/standard dose–response curves.^9^, ^45^ (details in Pliška and Krejčí^45^ accessible via ResearchGate portal, https://www.researchgate.net/publication/17259066).

### 2.2 Animals and tissue preparations

Female virgin Sprague–Dawley rats (body weight approximately 200 g) in natural oestrus (detected by vaginal smears) were sacrificed by decapitation, both uterus horns were dissected and the perimetrium was pulled off. Their middle sections (approximately 15 mm) were longitudinally cut into 3‐ to 4‐mm strips; the endometrium (the inward layer of the horns) was removed by scraping. In average, three strips of each animal (31 rats in total) were used in the experiments.

### 2.3 Experimental procedure

A stripe was mounted in an adapted annealed organ chamber (17 mm × 65 mm) with a ventilation inlet tube containing 10‐ml Munsick medium (see above) and attached to an isometric force transducer. The basic tension was adjusted to 10 ± 0.2 mN (1 gf, gram‐force). Measurements were carried out at 30°C under continuous ventilation with Carbogen gas (25% O_2_ + 5% CO_2_). The protocol of time–response measurements was presented in detail in our precedent communication.^22^ Each strip used for stimulation by a single peptide (for concentration of stimulating agents, cf. Table 2). Oil‐immersion and washout experiments were carried out on different strips from the same animal. Stimulation runs (two to four per strip) for each peptide were repeated on strips of different rat uteri. In order to avoid a presumed effect of tachyphylaxis, the duration of an experiment did not exceed 5 h. Number of strips used in oil‐immersion experiments is indicated in Table 2.

**TABLE 2:** Oxytocin analogues: Estimated rate constants *k*_*r*_, *κ* (min^−1^) and affinities in K^+^‐depolarizer rat myometrium

Peptide	Group^a^	No. of strips	Runs^b^	*κ*		*κ*_(*N*)_ − *κ*_(*D*)_ ^e^	*k*_*r*_ ^c^	Cumulative clearance *k*_*r*_ *+ κ* ^c^	*k*_*ρ*_ ^f^	K^+^‐depolarized myometrium p*C*_*E*_ ^g^
				Observed^c^	Predicted ^d^					
OXT	N	6	35	0.137 ± 0.025	0.133	0.088	0.137 ± 0.030	0.254 ± 0.028	0.250	7.25 ± 0.45
DOT	D	9	18	0.049 ± 0.021	0.037		0.149 ± 0.026	0.176 ± 0.024	0.148	8.28 ± 0.16
C_1_OT	N	2	8	0.162 ± 0.032	0.181	0.060	0.116 ± 0.041	0.272 ± 0.037	0.277	7.43 ± 0.41
DC_1_OT	D	4	6	0.102 ± 0.016	0.089		0.097 ± 0.025	0.160 ± 0.021	0.172	7.62 ± 0.24
C_1,6_OT	N	2	3	0.076 ± 0.005	0.096	*0.076*	0.194 ± 0.011	0.263 ± 0.009		8.76 ± 0.11
DC_1,6_OT^h^	D				≈0		*0.226*			4.94
AOT	N	2	5	0.118 ± 0.008	0.115	0.076	0.158 ± 0.020	0.278 ± 0.015		
DAOT	D	7	11	0.042 ± 0.015	0.029		0.139 ± 0.044	0.194 ± 0.033		6.68 ± 0.50
GOT	N	1	2	0.166	0.169	0.092	0.108	0.267		7.44 ± 0.44
DGOT	D	2	6	0.076 ± 0.018	0.084		0.107 ± 0.034	0.211 ± 0.027		8.36 ± 0.20
HOT	N	2	12	0.141 ± 0.008	0.137		0.139 ± 0.012	0.289 ± 0.010		5.41 ± 0.77
DC_6_OT	D	6	7	0.105 ± 0.008	0.108		0.080 ± 0.020	0.147 ± 0.015	0.127	5.41 ± 0.77
Pooled^i^	N		29	0.136 ± 0.028		0.079 ± 0.014	0.139 ± 0.031	0.266 ± 0.013		7.25 ± 0.45
	D		40	0.073 ± 0.039			0.126 ± 0.041	0.172 ± 0.026		8.28 ± 0.16

^a^ N: peptides carrying hydrophilic *N*‐terminal group NH_2_ or OH, D: deamino‐analogues.

^b^ Total number of computation runs (various estimation procedures and initial parameter estimates).

^c^ Arithmetic mean ± standard deviation (when no. of runs > 2). Dimension min^−1^.

^*d*^ Values recomputed from substituent contributions obtained by Free–Wilson analysis.

^e^ *κ*‐difference of coherent N & D pairs *κ*_*(N),*_ *κ*_*(D)*_ (reflects a fraction of the overall inactivation rate equivalent to the splitting of *C*‐terminal peptide bond). In italics: DC_1,6_OT estimate (cf. footnote d), not included in the mean value (i).

^f^ Affinity constants p*C*_*E*_ ≡ −log *C*_*E*_ estimated in this project. Arithmetic means ± standard deviations of (number of computed values: 3 to 15). Data obtained by Equation (12).

^g^ Not used in the present oil‐immersion experiments (presented are solely predicted values; cf. footnote d).

^h^ Mean ± standard deviation of values of N and D groups.

^i^ Means ± standard deviations of values in groups N & D resulting in individual computation runs (value in italics deleted)

**FIGURE 2:**
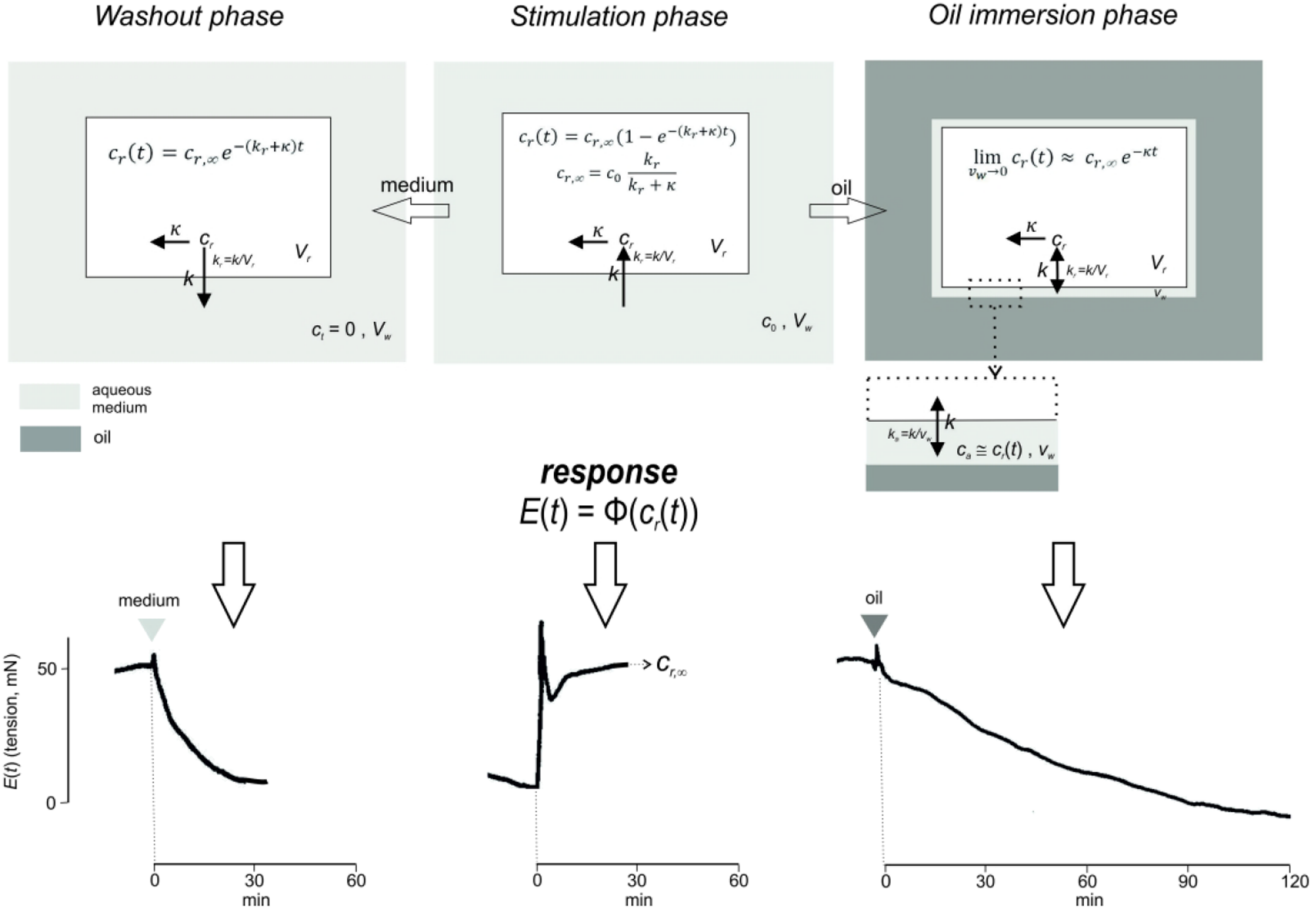
Phases of oil‐immersion experiment in the oxytocin stimulated myometrium: compartment model and clearance kinetics (upper panels). The compartment system of peptide distribution (upper panels) consists of the receptor compartment as a part of the interstitial space (white background) and the aqueous tissue medium (light grey), suppressed in the oil‐immersion phase by the mineral oil (dark grey). Upper part: stimulation phase (middle block), washout (left‐hand block), insertion of oil (right‐hand block; aqueous medium reduced from *V*_*w*_ to *v*_*w*_). The inserted block in the right‐hand panel depicts a magnified membrane section with the potential remnant of the aqueous medium after the displacement by oil. Vertical arrows between two compartments indicate directions of the peptide transport, horizontal arrows its irreversible clearance. State (and steady state) equations for the receptor compartment (time change of the peptide concentration *c*_*r*_) are indicated. Lower part: response profiles (isometric contraction) of the depolarized myometrium strip to oxytocin in the respective phase. *E*(*t*) is a (here nonspecified) time–response function Φ of *c*_*r*_(*t*)

Figure 2 shows in a synoptic form the experimental setup. Tonic contraction of the depolarized myometrium strips in a high potassium Ringer organ bath medium^46^ (concentration of Ca^2+^ and of Mg^2+^: 0.5 mM) was elicited by roughly equipotent concentrations of single peptides around their *D*_2_ values (the concentration causes the half of the maximally attainable contraction in oestrogen‐dominated myometrium). After reaching a steady‐state level (stimulation phase), the medium was exchanged by a low viscosity paraffin oil (oil‐immersion phase) or alternatively by the peptide‐free bath medium (washout phase). The time‐tension profiles of the strip were recorded by an isometric force transducer (Statham strain gauge UC3).

### 2.4 Evaluation routines

The software package Wolfram Mathematica™ (version 11.3) was employed for integration of differential equation systems describing the compartmented model (see below). Time‐tension profiles were digitized in regular time intervals (0.3 to 2 min, according to the total length of the decay record). SYSTAT (version 13.2, *NONLIN*, regression, descriptive statistics and testing routines), GraphPad Prism (version 8.3) and in part also MATLAB routines (version R2021a: interpolation, *interp1*) were used for parameter estimates by nonlinear regression routines, numerical operations in the Free–Wilson analysis (see below), descriptive statistics and statistical testing.

## 3 RESULTS

### 3.1 Kinetic analysis

#### 3.1.1 “Stopped‐transport” by oil‐immersion

The structure of the compartment system employed in oil‐immersion experiments was described in a block box form in our recent communication.^22^ We employed here its modified version focused on the comparison of clearance and transport rate constants (Figure 2). It consists virtually of two distribution spaces: the receptor compartment (subscript *r*; see Section 4) and the external aqueous tissue medium (*w*). The rate of mass transport of agents through the tissue‐medium interface (transport constants *k*_*r*_, *k*_*w*_) is directly proportional to the rate constant of diffusion *k* (dimension: volume/time) and inversely proportional to the respective compartment volume *V*_*r*_, *V*_*w*_: for the receptor compartment *k*_*r*_ = *k*/*V*_*r*_, for the medium *k*_*w*_ = *k/V*_*w*_. The constant *κ* relates to the (irreversible) clearance rate from the receptor compartment (dimension: time^−1^). The rate equations determining the time response of the peptide concentrations in the corresponding compartments (*c*_*r*_, *c*_*w*_) are then

(1a) $${\dot{c}}_{w}=-k\left(c_{w}-c_{r}\right)/V_{w},$$

(1b) $${\dot{c}}_{r}=k\left(c_{w}-c_{r}\right)/V_{r}-\kappa \;c_{r},$$

wherein dotted symbols denote first derivatives with respect to time *t*: $\dot{x}\equiv \frac{dx}{dt}$.

1. In the stimulation phase, concentration *c*_*w*_ is expected to be constant, *c*_*w*_ = *c*_0_ (and ${\dot{c}}_{w}=0$) within the time interval of stimulation; the integration of Equation 1b yields

(2) $$c_{r}\left(t\right)=c_{r,\infty }\left(1-e^{-\left(k_{r}+\kappa \right)t}\right),$$

where *c*_*r,∞*_ is a steady state value of *c*_*r*_,

(3) $$c_{r,\infty }\equiv \lim_{t\to \infty }c_{r}\left(t\right)=c_{0}\frac{k_{r}}{k_{r}+\kappa }.$$

- In the wash‐out decay phase, the concentration in the external medium (*c*_*w*_) is kept zero by a quick perifusion; *c*_*r,∞*_ is the initial value (*t* = 0), i.e., a *c*_*r*_ steady state value before termination of the stimulation phase,

(4) $$c_{r}\left(t\right)=c_{r,\infty }e^{-\left(k_{r}+\kappa \right)t}.$$

The sum *k*_*r*_ + *κ* stands for the cumulative clearance rate constant of the peptide in the receptor compartment.

- The insertion of oil (oil‐immersion decay phase) reduces the volume *V*_*w*_ to a small residual aqueous layer *v*_*w*_ around the tissue; obviously, *v*_*w*_ ≪ *V*_*w*_ and the corresponding modified mass transport rate constant $k_{w}^{\prime }=k/v_{w}$ is very large, $\lim_{v_{w}\to 0}k_{w}^{\prime }=\infty$. The integration of the homogenous linear system (1) brings forth a sum of two exponentials

(5) $$c_{r}\left(t\right)=\frac{c_{r,\infty }}{\lambda_{2}-\lambda_{1}}\left(\lambda_{2}e^{\lambda_{1}t}-\lambda_{1}e^{\lambda_{2}t}\right),$$

with exponential coefficients resulting from the roots *λ* of its characteristic polynomial,

(6a) $$\lambda^{2}+\left(\kappa +k_{r}+k_{w}^{\prime }\;\right)\lambda +\kappa \;k_{w}^{\prime }=0.$$

Supposed that the volume *v*_*w*_ is very small, $k_{w}^{\prime }\gg \kappa +k_{r}$, and the characteristic polynomial can be reduced to

(6b) $$\lambda^{2}+k_{w}^{\prime }\lambda +\kappa \;k_{w}^{\prime }=0.$$

The resulting exponential coefficients are

(7) $$\lambda_{1,2}=0.5\left(-k_{w}^{\prime }\mp \sqrt{{k_{w}^{\prime }}^{2}-4\;\kappa \;k_{w}^{\prime }}\right).$$

For small *κ* values (*κ* ≪ $k_{w}^{\prime }$), the Maclaurin expansion for the variable *κ* (or alternatively *v*_*w*_) about its zero value yields linear terms of the coefficients *λ*,

(8a) $$\lambda_{1}\approx -k_{w}^{\prime }+\kappa \approx -k_{w}^{\prime },$$

(8b) $$\lambda_{2}\approx -\kappa .$$

The rate equation corresponding to Equation 5 runs

(9a) $$c_{r}\left(t\right)=\frac{c_{r,\infty }}{k_{w}^{\prime }-\kappa }\left(k_{w}^{\prime }e^{-\kappa \;t}-\kappa \;e^{-k_{w}^{\prime }t}\;\right).$$

Thus, still assuming that $k_{w}^{\prime }\gg \kappa$, the concentration *c*_*r*_(*t*) in the oil‐immersion phase follows approximately a single exponential course,

(9b) $$c_{r}\left(t\right)\approx c_{r,\infty }e^{-\kappa \;t}.$$

#### 3.1.2 Response kinetics: Combined steady state with exponential terms

The intrinsic relaxation dynamics of the stimulated tissue is another factor of the muscle state change, potentially overlapping with the “pure” drug effect. A recent kinetic analysis of the uterotonic response to oxytocin and deaminooxytocin^22^ indicates that such additional dynamic processes in myometrium are not rate determining. The rectangular hyperbolic function commonly used to approximate steady state dose–response relationships may then describe a transient response *E*(*t*) at a concentration *c*_*r*_(*t*) in a decay phase,

(10) $$E\left(t\right)=E_{m}\frac{\;c_{r}\left(t\right)}{C_{E}+c_{r}\left(t\right)},$$

wherein *C*_*E*_ stands for an “intrinsic affinity” constant in the K^+^‐depolarized state (in mol L^−1^), formally expressing a concentration *c*_*r*_ that elicits the half‐maximal response, that is, *E*(*t*) = 0.5 *E*_*m*_. for *c*_*r*_ = *C*_*E*_. The response *E*(*t*) relative to its initial value (at the time *t* = 0; see above) is then

(11) $$\varepsilon \left(t\right)\equiv \frac{E\left(t\right)}{E\left(0\right)}=\left(1+\frac{C_{E}}{c_{r,\infty }}\right)\frac{\;c_{r}\left(t\right)}{C_{E}+c_{r}\left(t\right)}.$$

The concentration functions *c*_*r*_(*t*) for the washout or the oil‐immersion phase, respectively, consist of a common pre‐exponential constant *c*_*r*,∞_ and a time dependent exponential term *τ*(t) of Equations 4, 9a and 9b,

(12a) $$c_{r}\left(t\right)=c_{r,\infty }\tau \left(t\right).$$

Then,

(12b) $$\varepsilon \left(t\right)=\frac{1+C_{E}^{*}}{\tau \left(t\right)+C_{E}^{*}\;}\tau \left(t\right),$$

where

(12c) $$C_{E}^{*}=\frac{C_{E}}{c_{r,\infty }}.$$

#### 3.1.3 Numeric analysis of time–response profiles

A nonlinear regression analysis of the time–response data using the Gauss–Newton iteration routine was applied to Equation (12b) combined with the exponential terms *τ*(t) of Equations 4, 9a and 9b. Concomitantly with the kinetic analysis, Equation 12 yields estimates of the intrinsic affinity *C*_*E*_ (mol  L^−1^) from the fitted constant $C_{E}^{*}\;$ (Equation 12c) and rate constants *k*_*r*_ and *κ*,

(13) $$C_{E}=C_{E}^{*}\;c_{0}\frac{k_{r}}{k_{r}+\kappa },$$

provided, however, that the steady‐state receptor ligand is sufficiently promptly achieved at each time point; *c*_0_ is the concentration in the bath medium (stimulating phase; cf. text at Equation 2). Resulting *C*_*E*_ values for uterotonic response of oxytocic peptides in the K^+^‐depolarized myometrium are added to Table 2. The mean ratio *C*_*E*_/*D*_2_ (*D*_2_ derived by conversion of in vitro IU data in Table 1, cf. chapter 2.2) over all listed peptides, 26.2 ± 5.6 (11 values), indicates a more than 20‐times decrease of myometrium sensitivity owing to depolarization and supports sporadic literature values—between 10 and 20.^47^, ^48^

Computations were carried out in several runs under changing initial conditions and/or using data in varying time intervals. Asymptotic standard errors (ASE)^49^ of parameter estimates in a particular regression run were taken as a “goodness of fit” criterion: estimates reaching the ratio *parameter*/ASE lower than 5 were not considered in further statistical evaluations. The criteria for *κ*, *k*_*r*_ for accepted estimates were in the range of 15 and 75. A numeric solution of the strongly nonlinear expression 12b commonly requires about 25 iteration steps. Loss functions in the optimization of such multiparametric expressions display frequently several local minima and hence may lead to different sets of parameter estimates, depending on the ranges of initial iteration values for the model parameters. The absolute minimum does not necessarily warrant a physically relevant solution; this requires setting the initial values within the assumed ranges of their physical relevance. Therefore, single decay profiles were evaluated under varying computational conditions (initial values of iteration, critical limits of the used loss function; using values from constrained time intervals; etc.); the arithmetic mean of the adjusted estimates was attributed to the respective rate constant value.

### 3.2 Kinetic and affinity data: Statistical conclusions

#### 3.2.1 Descriptive statistics

**FIGURE 3:**
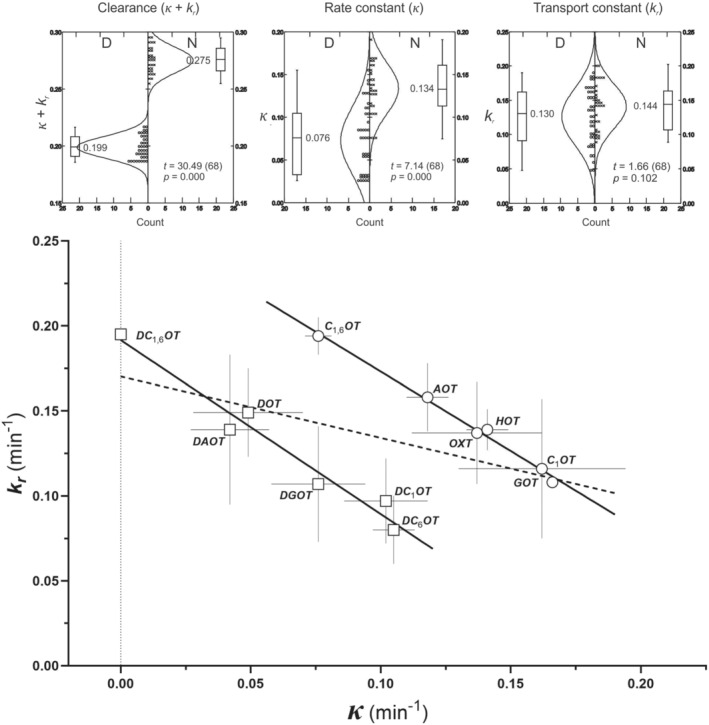
Relationship between clearance (*κ*) and transport rate constant (*k*_*r*_) within N (circles) and D (squares) analogue groups. Values of constants in Table 2. Arithmetic means and standard deviations (error bars). Correlations within the groups are highly significant. Upper panels: box plots of pooled *κ*, *k*_*r*_, and (*κ* + *k*_*r*_) data. Each box represents the interquartile range (1st to 3rd quartile), the median (cross‐line in the box), highest and lowest values (bars); *t* and *p* values indicate the significance of the N‐D difference (*t*‐test)

Table 2 reveals mean values of the respective rate constants obtained in single experiments. Due to the potential multimodality of the loss function mentioned above, the set of estimated rate constants *κ* and *k*_*r*_ may contain values that are out of the expected physical range. Estimates tainted by high asymptotic errors were sorted out already within the computation routine, and those lying outside of the 95% group confidence limits around the arithmetic mean were taken as outliers and not considered in further evaluations.^50^ Resulting values of the two rate constants for single peptides are of the same order of magnitude (cf. Table 2), within the limits 0 to 0.19 for *κ* and 0.10 to 0.20 for *k*_*r*_. On a first examination, mean *κ* values in the groups of peptides with a polar (group N) and a neutral *Ν*‐terminus (D) are obviously different, whereas the transport constants *k*_*r*_ are rather monotonically distributed over the whole data set. Box plots in Figure 3 (upper panel) show the data distributions and the results of a two‐sample *t*‐test of N‐D differences: significant difference exists in *κ*‐ and aggregate clearance values (*κ* + *k*_*r*_), but not in transport constants *k*_*r*_.

Relationships between individual estimates of the rate constants *κ* and *k*_*r*_ were analysed by a linear regression analysis (totally 80 values) after deleting evidently declining cases. The employed computation procedure^51^ enables to detect further outlying values and calculate stochastic criteria (correlation coefficients and their probabilities *p*) of the full and the reduced sets. As expected, the two constants, derived from the related exponential coefficients in Equations 4, 9a and 9b, are inversely correlated (correlation coefficient *r* =  −0.91, *p* = 0.000). Already at the first inspection, the data in the D and N arrays are clearly separated and display distinct correlations, not differing in slopes (two‐tailed *t*‐test: *p* = 0.461) but clearly shifted along the abscissa (*p* < 0.0001). The significantly different course of the two lines validates, in addition to the box plot in Figure 3 (lower panel), the clear‐cut pharmacokinetic differences between oxytocin analogues with polar and with rather hydrophobic *N*‐terminus.

As expected, computed values of $k_{w}^{\prime }$ in the oil‐immersion phase (Equation 9a) are very high, usually approaching limit values, $k_{w}^{\prime }$ > 10^9^. This confirms the assumption that the volume of the residual aqueous layer in the oil‐immersion phase (*v*_*w*_) is very small and negligible and thus approves the use of single exponential time function *c*_*r*_(*t*) (Equation 9b) as a numerically simpler computational alternative. Moreover, this operational negligibility validates via *facti* the suggested compartment model.

The applied optimization routines yield consistent values of *κ* and the cumulative clearance (*κ + k*_*r*_). The same is true also for the constant *k*_*r*_, although a somewhat higher scatter might actually be anticipated due to its dependence on *V*_*r*_ (assumed to vary in various tissue preparations). However, there remains a certain casualness in the formulation of *k*_*r*_ values since they are, for experimental reasons, derived from independently combined pairs of data from oil‐immersion and washout phases: the two cannot frequently be obtained in a single experiment, because tachyphylaxis restricts a longer use of the same uterus strip. In such instances, *κ* and (*κ + k*_*r*_) data pairs were combined randomly. Possible linear relationships between paired model descriptors—rate (*κ, k*_*r*_) and affinity descriptors (p*C*_*E*_: the negative decimal logarithm of *C*_*E*_)—are fully insignificant: the Pearson correlation coefficients (absolute values) lie between 0.08 and 0.26 (uncorrected probabilities for *n* = 58, *p* > 0.30).

#### 3.2.2 Relationship between in vivo and ex vivo clearance descriptors

**FIGURE 4:**
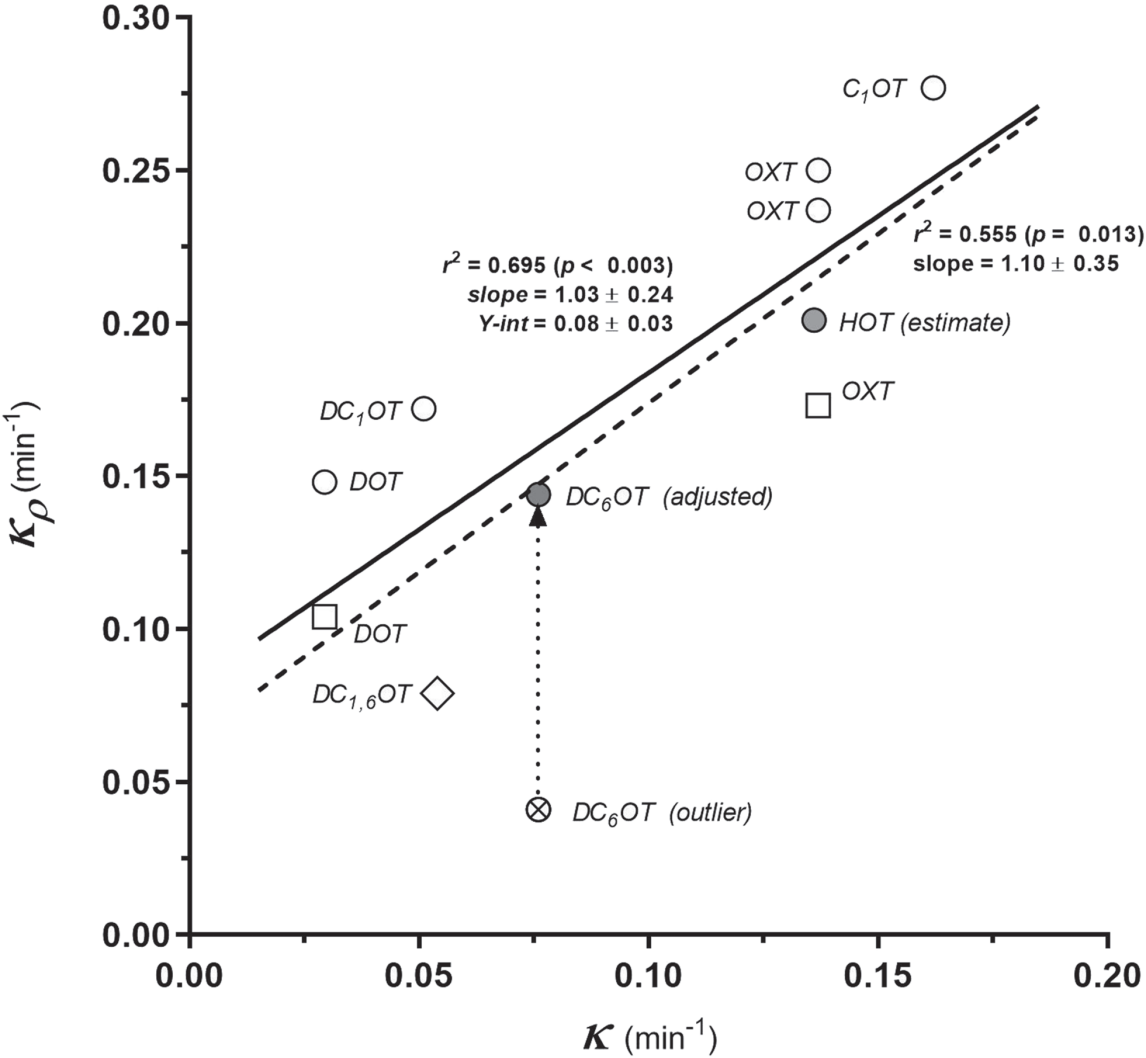
Relationship between ex situ (*κ*) and in vivo (*k*_*ρ*_) clearance rate constants (arithmetic means). Circles: reported *k*_*ρ*_ (reference s. Table 1), squares: *k*_*ρ*_ values from our laboratory. Grey circles: adjusted or extrapolated values (s. text); the cross‐circle (DC_6_OT) is a value reported in the literature and identified here as an outlier, the arrow indicates corrected value. Full line: regression over nonoutlying values, broken line: reported values including the outlier. Significance criteria are shown at each line

The in vivo total decay rate constants *k*_*ρ*_ for the uterotonic response are reported in several communications and listed in Tables 1 and 2. They reflect, in analogy to the cumulative constants *κ + k*_*r*_, clearance processes in the receptor compartment within the framework of the intact organism, that is, in a native (nondepolarized) state of myometrium. Their numeric values, assessed from half‐lives after cessation of a prolonged infusion^3^ or from the slopes of the dose–response curves^9^ after doubling the stimulation doses,^7^, ^52^ were estimated earlier in several laboratories (cf. Table 1). Differences of mean values *k*_*ρ*_ and (*κ + k*_*r*_), tested by the paired *t*‐test, are insignificant (*p* = 0.090), although displaying a slight, likely most experimentally caused, propensity toward *k*_*ρ*_ < (*κ + k*_*r*_). This supports the assumption that the kinetics of the peptide clearance processes in rat depolarized and “native” (nondepolarized) uterus do not differ. Hence, the time profiles in the—methodically preferable—depolarization state reflect the in vivo kinetics of oxytocin‐like peptides in the OXTR compartment sufficiently well. As intuitively anticipated, a correlation between the data pairs *κ*, *k*_*ρ*_ (Tables 1 and 2; the *κ* value of DC_1,6_OT is a prediction by the Free–Wilson analysis; see below) are closely correlated (Figure 4): the slope of the regression line only insignificantly differs from unit. The *k*_*ρ*_ value of DC_6_OT reported in the literature^4^ seems to strikingly decline from the regression line (circle cross in Figure 4). The adjusted value (*k*_*ρ*_ = 0.144) was obtained by linear interpolation using the MATLAB function *interp1*, and we suggest considering this value as a more probable one. However, the replacement of the deviating value does not improve the correlation significantly (two‐sample *z* test after Fisher's *r* to *z* transformation), but it yields a “sharper” value of the slope (1.066 ± 0.262). No *k*_*ρ*_ value for HOT has been reported for uterotonic response, but its reliable value could be approximately estimated from the half‐lives of OXT and HOT obtained for antidiuretic response.^6^

### 3.3 Segment contributions to rate constants *κ* and *k*_*r*_: Free–Wilson analysis

Under the assumption that distinct sites of the peptide chain potentially exposed to enzymatic attacks participate additively in the overall inactivation rate, the constant *κ*_*i*_ of an *i*th peptide is expressed as

(14a) $$\kappa_{i}=\kappa_{0}+\sum_{j}\sum_{g}\xi_{\mathrm{ijg}}\phi_{\mathrm{jg}},$$

where *κ*_0_ stands for a backbone contribution (regarded as a common invariable molecular segment within the investigated set of peptides, in our case that of oxytocin) and *ϕ*_*jg*_ for a segment contribution of the *g* substituent group in the background position *j* to the resulting rate constant. The same approach applies for the transport constant *k*_*r*_. *ξ*_*ijg*_ is a local dummy parameter: *ξ*_*ijg*_ = 1 if the *j*,*g* segment is present in the particular *i* peptide, *ξ*_*ijg*_ = 0 otherwise. The method suggested by Free and Wilson^39^ offers a solution of the equation system 13, extended of standardized sums of *ξ*_*jg*_ at single positions *j* by setting them to zero,

(14b) $$0=\sum_{i}\sum_{g}\xi_{\mathrm{ijg}}.$$

**FIGURE 5:**
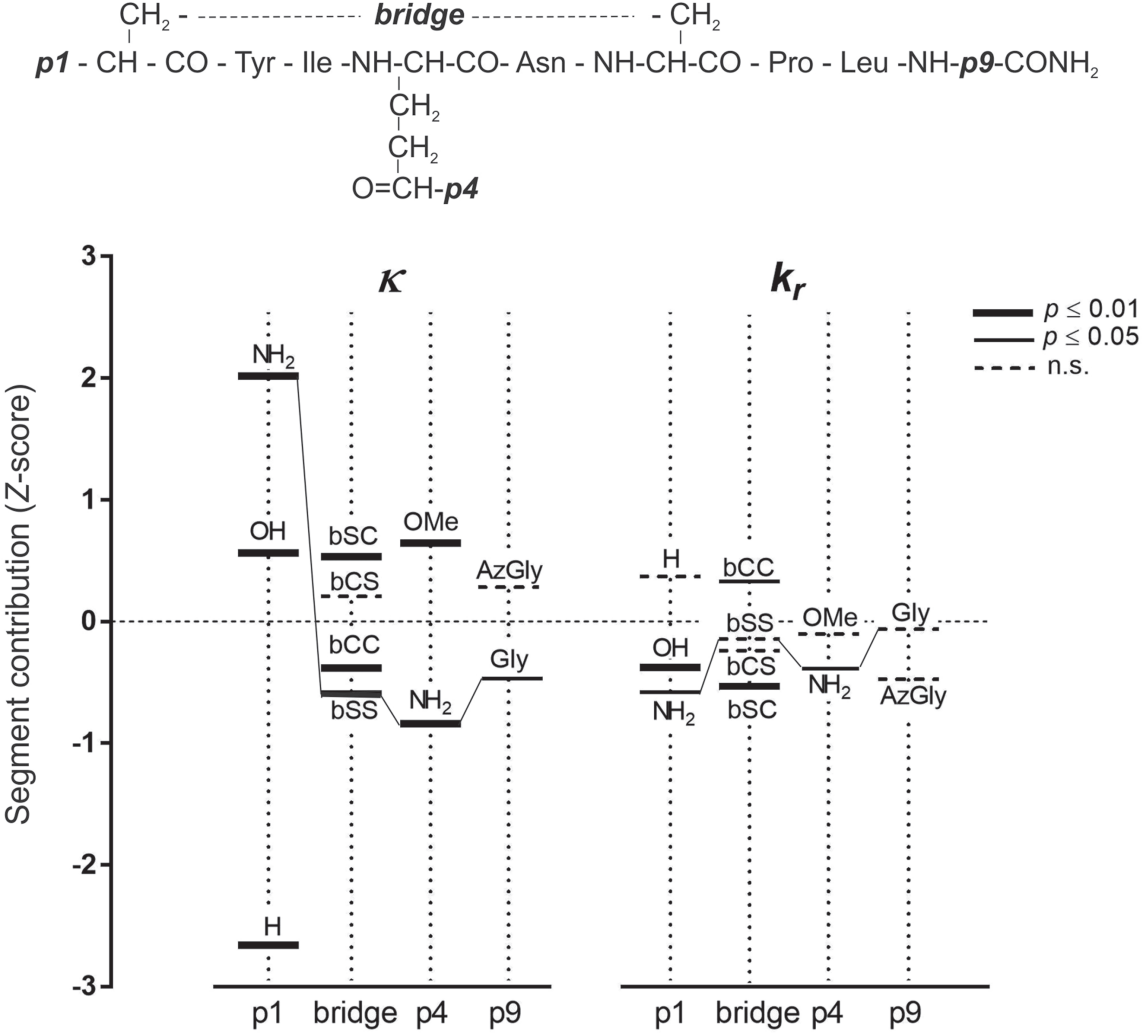
Segment analysis of rate constants *κ* and *k*_*r*_. *Z*‐transformed segment contributions to the standardized backbone (*Z*‐score = 0, horizontal broken line) for four sites of the oxytocin chain (upper part). Significance levels estimated by the regression analysis are marked with different horizontal bars shown in the inserted legend (right‐hand). Connecting lines designate segments in the oxytocin chain. Symbols (in addition to common chemical nomenclature): modified disulphide bridge in dicarba‐analogues – bCC, in carba^1^‐analogues – bCS, in carba^6^‐analogues – bSC, in intact S‐S bridge – bSS; AzGly – hydrazinoacetyl (‐NH‐NH‐CH_2_‐CO‐)

The *ϕ*_*jg*_ values follow from a solution of the resulting linear system, here by the general linear model routine of SYSTAT (comprising the constant term). A regression analysis for the sets *κ* and *k*_*r*_ indicates in both cases a significant linearity (*p* < 0.0001); this rules out any mutual interdependencies of individual segment contributions *ϕ*_*jg*_ (the regression coefficients between them are mostly fully insignificant). Within the analysed group, the estimated *ϕ*_*jg*_ values allow to derive predicted *κ* and *k*_*r*_ descriptors of the analysed peptides, and occasionally to obtain predictions for missing values of additional peptides (in our set for DC_1,6_OT). Corresponding predicted *κ*‐descriptors are included in Table 2. At first sight, the correlation between observed and predicted values is tight and confirms the additivity of segment contributions to *κ* (Equation 14): the regression coefficient is close to unit (1.015 ± 0.031). Standardized (*Z*‐transformed) *ϕ* descriptors for four investigated sites are shown in the segment diagram of Figure 5. In this form, the contributions relate to the backbone value, which appears as a zero point in the chart (horizontal dotted line). Values of descriptors *κ* (left‐hand side) are predominantly determined by substituents in position 1 (p1; see Figure 1) that are in all instances highly significant (*p* ≤ 0.01): polar groups (NH_2_ or OH) contribute positively to *κ*, their omission leads to significantly more negative *ϕ* descriptors. Significant (*p* ≤ 0.05), but numerically less relevant for the final *κ* values, are the substituents in the intramolecular ‐S‐S‐ bridge between positions 1–6 (“bridge” in Figure 5). As compared with the native ‐S‐S‐ bond, the substitution of individual sulphur atoms with the CH_2_ group, assumed to protect the cyclic structure of oxytocin against disulphide reduction, results surprisingly even in an increase of the *κ* values. The cleavage of the *N*‐terminal Cys‐Tyr bond by other aminopeptidases while retaining the ring structure seems to be the likelier inactivation process. Thus, the integrity of the peptide ring structure plays obviously no role in the overall clearance process. A substitution of the peptide bond in the *C‐*terminal position 9 (p9) has virtually no positive effect on the peptide stability in the receptor compartment and, consequently, on the response dynamics.

Effects of the reported site substituents on the transport constant *k*_*r*_ (right‐hand panel in Figure 5) are, in general, not significant. The only noteworthy exceptions are hydrophilic substituents in positions 1 (OH and NH_2_ groups on C^α^ of 1‐hemicystin residue) and 4 (NH_2_ of glutamine). Their overall effect on *k*_*r*_ is, however, not overwhelming.

### 3.4 Specific case of GOT and DGOT: Fragmentation of rate constants

An introduction of the glutamic acid *δ*‐methyl ester in position 4 results in a significantly higher positive contribution to the *κ* values (Table 2, Figure 5). This additional step is obviously a hydrolysis of the ester bond which seems to be a fast inactivation process.^10^ The hydrolysis (most likely an enzymatic one) would lead to metabolites with a residual uterotonic activity: the peptide corresponding to oxytocin, [Glu^4^]OXT, is rather low active (≈1.5 IU^53^), whereas the deamino‐analogue, [Glu^4^]DOT,§ possesses a significant activity (13.3 IU^54^). The recorded superimposed time–response *ε*(*t*) comprises activities of the analogue itself (*α*_*A*_) plus its corresponding metabolite [Glu^4^]OXT or [Glu^4^]DOT (*α*_*M*_),

(15) $$\varepsilon \left(t\right)=\alpha_{A}\varepsilon_{A}\left(t\right)+\alpha_{M}\varepsilon_{M}\left(t\right).$$

This is an alternative to model represented by Equation 12b, where*ε*_*A*_(*t*), *ε*_*M*_(*t*) are response fractions of the respective primary agents and of its active metabolite(s)

(16a) $${\dot{\varepsilon }}_{A}\left(t\right)=-\left(\kappa_{c}+\kappa_{A}\right)\varepsilon_{A}\left(t\right),$$

(16b) $${\dot{\varepsilon }}_{M}\left(t\right)=\kappa_{c}\varepsilon_{A}\left(t\right)-\kappa_{M}\varepsilon_{M}\left(t\right).$$

**FIGURE 6:**
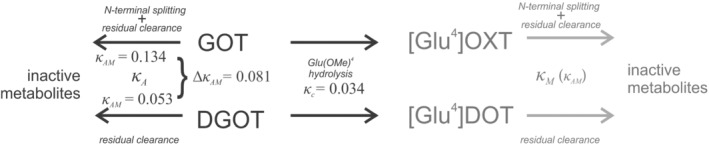
Fragmented inactivation model: 4‐substituted oxytocin analogues (GOT & DGOT). The rate constant *κ*_*c*_ stands for the conversion of [Glu (OMe)^4^]‐analogue of oxytocin and deaminooxytocin to [Glu^4^]‐peptides, *κ*_*A*_ and *κ*_*M*_ (assumption: *κ*_*A*_ ≈ *κ*_*M*_ = *κ*_*AM*_) are clearance rate constants of the analogues (left‐hand side) and their metabolites (right‐hand side, symbols in gray), respectively. Δ*κ*_*AM*_ stands for the difference between amino‐ and deamino‐analogues (rate constant of the assumed *N*‐terminal splitting)

Figure 6 depicts the proposed reaction scheme. The rate constant *κ*_*c*_ relates to the conversion of the primary peptide (A) into its biologically active metabolite (M), the rate constants *κ*_*A*_ and *κ*_*M*_ to the final irreversible inactivation of A and M. Under the simplifying assumption that the rates of irreversible A and M conversions are roughly identical, *κ*_*A*_ ≈ *κ*_*M*_ ≡ *κ*_*AM*_, the solution of the system (16) inserted into the “conservation” Equation 15 runs

(17a) $$\varepsilon \left(t\right)=\left(1-\Delta_{\alpha }\right)e^{-\left(\kappa_{\mathrm{AM}}+\kappa_{c}\right)t}+\Delta_{\alpha }e^{-\kappa_{\mathrm{AM}}t},$$

or, when the predetermined constant *κ* (Table 2, *κ* = *κ*_*AM*_ *+ κ*_*c*_) is inserted as a fixed parameter,

(17b) $$\varepsilon \left(t\right)=\left(1-\Delta_{\alpha }\right)e^{-\kappa \;t}+\Delta_{\alpha }e^{-\left(\kappa -\kappa_{c}\right)t}.$$

*Δ*_*α*_ = *α*_*M*_/*α*_*A*_ is the standardised biological activity (e.g., in IU/mg). Exponential constants for GOT and DGOT were obtained by the nonlinear regression analysis using numeric procedures described above and are summarized in Table 3. Obviously, nonlinear regression procedures applied to Equation 17b minimize the asymptotic errors of *κ*_*c*_ but, sensu stricto, the derived *κ*_*MA*_ estimates obtained as *κ* − *κ*_*c*_ depend of previous computations and thus, are not fully independent; however, the two variants of Equation 17 generate congruent parameter values. Irrespective of the use of differing *α* values reported from different laboratories^5^, ^10^ (see Table 1), the regression yields for both peptides consistent values of the rate constant *κ*_*c*_ of the implicit ester hydrolysis constant (A → M conversion), *κ*_*c*_ = 0.034. Ester hydrolysis is then no more projected in the computed rate constant *κ*_*AM*_; therefore, its value for DGOT (0.053) roughly represents other, less specific inactivation processes involved in the enzymatic splitting of the basic peptide chain of these two oxytocin analogues. This inductively applies for any pair of analogues with an equal residual structure. Moreover, the difference of *κ*_*AM*_ in the N (GOT) and D (DGOT) groups, Δ*κ*_*AM*_ = *κ*_*AM*(*N*)_ − *κ*_*AM*(*D*)_ ≈ 0.08, confirms the rate constant of *N*‐terminal splitting obtained from the N‐D difference as *κ*_(*N*)_ − *κ*_(*D*)_ in Table 2. In this way, the suggested analysis of these two unique oxytocin analogues enables a closer look at the weight proportion of distinct processes participating in the clearance in the receptor compartment.

**TABLE 3:** GOT and DGOT: Fragmented rate constants

Peptide	Uterotonic activity (IU/mg)		Fragments of *κ*			Estimates of *κ*		
	Peptide^a^	[Glu^4^]‐analogue	*κ*_*c*_ ^b^ (min^−1^)	*κ*_*AM*_ ^b^ ^,^ ^c^ (min^−1^)	*Δκ*_*AM*_ ^d^ (min^−1^)	*κ*_*AM*_ + *κ*_*c*_	*κ* ^e^	
GOT	10.5; 18	1.5 ^f^	0.033 ± 0.012 (22) <0.028; 0.038>	0.134 ± 0.025 (12) <0.132; 0.135>	**0.081**	0.167	0.166	*0.169*
DGOT	21.4; 43	13.3^g^	0.032 ± 0.011 (14) <0.025; 0.038>	**0.053** ± 0.014 (14) <0.045; 0.061>		0.085	0.076	*0.084*
Pooled GOT + DGOT			**0.034** ± 0.011^h^ (36) <0.030; 0.037>					

^a^ References: (1st values),^10^ (2nd values).^11^ Values in international units per mg substance.

^b^ Arithmetic means ± standard deviations, number of computation runs in parentheses. <cl1; cl2 > stands for 95% confidence limits (cl1—lower, cl2—upper). Bold: expected value of glutamic acid‐δ‐methylester hydrolysis.

^c^ Rate constant of residual clearance (bold); expected value of *C*‐terminal splitting.

^d^ Difference of mean values *Δκ*_*AM*_ = *κ*_*AM*_ (GOT)—*κ*_*AM*_ (DGOT). Bold: expected value of rate constant of *N*‐terminal (aminopeptidase) splitting.

^e^ Rate constants obtained by exponential analysis (Table 2). In italics: values predicted by the segment analysis.

^f^ Photaki and du Vigneaud.^53^

^g^ Ferrier and Branda.^54^

^h^ Rate constant of ester hydrolysis [Glu(OMe)^4^]‐X $\overset{\kappa_{c}}{\to }\;$ [Glu^4^]‐X (bold).

## 4 DISCUSSION

### 4.1 Pharmacokinetic aspects

The clearance rate constants for the receptor compartment were assessed from the decay phase of the time–response profile using the combined exponential and hyperbolic response functions (Equation 11). An asymptotic relationship between the response and the time values of the concentration turned to be the most simple and the most suitable time–response function. An extension of the orthogonal hyperbola in our computations (Equation 10) to rational fractions (Hill or Adair equations) is not pragmatic: formulas used for numerical solutions are frequently overparametrized. Moreover, the Hill power coefficient for oxytocin analogues, estimated for oxytocin in in vitro dose–response measurements in several laboratories, is close to unit (the mean ± standard error of available reported data for oxytocin is 1.10 ± 0.13, values for five analogues investigated in this study lie between 0.71 and 1.29,^55^ with a mean of 0.98 ± 0.22).

The suggested compartment structure itself and the employed methodical approach require some additional comments. First, a receptor compartment is currently defined as a structural unit in which the concentration of a stimulating agent regulates a specified response.^22^, ^23^, ^24^ In this sense, it belongs to the pharmacologically formulated group of “fluid compartments.” These are conventionally considered as space elements of biological entities (organisms, tissues, cells, etc.) containing a defined agent in a homogenous concentration environment. The intracellular concentrations of solutes in such a bordered space are indeed not uniform, be it due to the inhomogeneity of the extracellular space medium, or due to the occurrence of the stimulating agents in several thermodynamic states (for instance, when bound in the vicinity of the receptor to additional macromolecular carriers). In the case of membrane‐bound receptors, the ligand–receptor interaction space is limited to the interstitial space close to the outer part of the cell membrane, which appears to be very narrow. Its topology in rat myometrium based on histometric data has been discussed in more detail in our previous communication.^22^ The concentration gradient of an amphiphilic substance—like peptides—near a biological membrane follows the Gouy–Chapman particle distribution around the negatively charged membrane surface^56^ and causes an inhomogeneity of the interstitium just on the ligand–receptor interaction site. Besides, capillary (or other hydrodynamic) forces may influence accumulation and depletion of particles within such a space. All this makes the effective concentration in the close proximity of the receptor site (*c*_*r*_), presently inaccessible to a direct assessment, difficult to express. Nevertheless, its apparent value can be expressed indirectly, as a concentration that elicits a measurable response. As follows from the analytical treatment of Equations (1) to (9), *c*_*r*_ is a function of the concentration in the external aqueous medium (*c*_0_), the clearance rate (*κ*) and the rate peptide transport (*k*_*r*_) in the steady state

(18) $$c_{r}=c_{0}P_{r/w}\frac{k_{r}}{k_{r}+\kappa },$$

where *P*_*r/w*_ is a steady state partition coefficient of the stimulating agents between receptor and the adjacent extrinsic compartment, defined as a time invariable ratio of free concentrations *c*_*r*_/*c*_*w*_. As far as no extensive barriers occur, *P*_*r/w*_ roughly equals unit.^22^ This may allow defining the receptor compartment operationally as a hypothetic extracellular space of a concentration *c*_*r*_ in which the ligand directly communicates with the respective membrane bound receptors. Despite a certain lack of sharpness, this concept may turn useful for kinetic investigations of drug kinetics in target tissues, as well as in further pharmacological considerations.

Second, besides the transport and clearance processes, the time course of a response to a drug stimulation can possibly be influenced by the kinetics of drug–receptor interaction, and/or by the internal contraction‐relaxation dynamics of the responding smooth muscle tissue. As for oxytocin, the influence of the receptor kinetics was investigated in more detail in our previous report, by using *k*_on_, *k*_off_ data for binding of oxytocin to myometrium membranes in in vitro preparations reported by various laboratories (summary in Pliska and Jutz^22^). Although the dissociation rate constant *k*_off_ is low (0.017 to 0.27 min^−1^), the estimated “formal” receptor concentration in the receptor compartment is high (for a concentration corresponding to *D*_2_ roughly 7 × 10^−6^ mol L^−1^) and thus, its effect on the oxytocin displacement rate is negligible. The spontaneous relaxation of the contracted myometrium cells succeeding the ligand dissociation from its receptor was described in the same communication as rather quick: the corresponding rate constant reached case dependent values around 1.5 min^−1^. These processes obviously played only an insignificant role in the experiments presented here.

Finally, the question arises as to whether the clearance and transport descriptors recorded in the specific instance of a depolarised smooth muscle apply also for its “native” (polarised cell membrane) state. For investigations of biochemical/biophysical processes in the receptor compartment, the depolarized state offers the experimental advantage of yielding smooth time–response data for the tonic component in the decay phase. High K^+^/Na^+^ ratio decreases the sensitivity of the contractile apparatus of myometrium cells by a factor of about 25 (Table 2) but does not substantially influence the clearance and the transport rate of the stimulating substance: the difference between cumulative clearance rate constants (estimated in the depolarized state) and response decay rate constants *κ*_*ρ*_ (native state) is insignificant. Moreover, the linear correlation between the ex vivo (*κ)* and in vivo clearance constants (*κ*_*ρ*_) (cf. Figure 4) provides a circumstantial support for the thesis that constants *κ* stand for irreversible (enzymatic) clearance processes in both in vivo and ex vivo systems.

### 4.2 Rate constants of the clearance processes: Biochemical traits

The enzymatic clearance rate *κ* in Table 2 displays conspicuous differences among oxytocin analogues used in this study as “enzyme” probes. The differences consist particularly in their belonging to the N and D groups: the *κ* values of the D group are distinctly lower. This is evident from the box plot in Figure 3 (upper panel) and, as mentioned above, from the *κ*, *k*_*r*_ relationship (lower panel). In addition, the Free–Wilson analysis (Figure 5) reveals that the most significant contributions to the *κ* values are linked to the status of the *N*‐terminal substituents: the NH_2_ and (somewhat less) the OH group exercise a positive, the proton (H) a negative effect upon *κ*. It confirms the earlier conclusions that the *N*‐terminal splitting by an aminopeptidase is the major component of the oxytocin‐type peptides clearance in the OXTR receptor compartment.

The dominating aminopeptidase splitting is indeed not the *only* one of the clearance processes. The segment contribution analysis suggests furthermore that also both carba^1^‐ and carba^6^‐bridges may enforce the clearance rate, although an experimental evidence for an ‐S‐S‐ role in oxytocin peptide inactivation by subcellular preparations is ambiguous. Singular enzymatic pathways may additionally occur in specific instances. Such were in our experiments analogues substituted by glutamic acid *δ*‐methyl esters in position 4 (GOT and in particular DGOT), where the unexpectedly low half‐life (high *κ*) indicates the occurrence of a significant supplementary rate component in their clearance. Although a direct biochemical evidence is lacking, such an additional step seems to be in the given case a hydrolytic splitting of the *δ*‐methyl ester, most likely an additional fast enzymatic process.

*C*‐terminal splitting of the oxytocin‐like peptides was considered as another possible clearance component. The rate constants *κ* of deamino‐analogues (Table 2) and the rate of residual clearance *κ*_*AM*_ in the case of DGOT (Table 3, Figure 6) suggest that this splitting may account for about 30% to 45% of the total clearance. Nevertheless, the available evidence appears somewhat ambiguous. In vitro hydrolysis of the Leu^8^‐GlyNH_2_^9^ ^34^, ^35^, ^57^ and (concomitantly or alternatively) Pro^7^‐Leu^8^ bond^37^, ^58^ was detected in various tissue homogenates (human uterus, toad bladder, kidney). The latter bond is also cleaved by purified bovine chymotrypsin.^59^ However, analogues substituted in position 9 by azaglycine—AOT and DAOT—reveal only slightly lower clearance rate constants *κ* compared with their 9‐glycine‐containing counterparts OXT and DOT (Figure  5). One can infer that also their clearances do not differ substantially from each other, and that the Gly → azaglycine substitution in position 9 does not exercise any appreciable inactivation protection. The insignificant difference of the two substituent segment contributions in position 9 (Figure 5) may allow for two explanations. Firstly, that a splitting of the *C‐*terminal bond Leu^8^‐GlyNH_2_^9^ in the myometrium does not regularly occur, although it was observed in homogenized tissue.^34^, ^35^, ^57^ Secondly, that the pseudo‐peptide bond leucine‐azaglycine (hydrazinecarboxylic acid, NH_2_‐NH‐CO_2_H) might potentially be cleaved at this structural site by an—not yet specified ‐ enzyme present in the myometrium tissue. This would explain small positive deviations of *κ* values compared with oxytocin. The other possible way, the post‐proline hydrolysis, appears even more dubious. Although, as mentioned, it was detected in vitro in human uterus homogenates, an ex vivo half‐life of a putatively protected analogue [7‐glycine]‐oxytocin^60^ paradoxically displays a considerably shorter half‐life of uterotonic response as compared with oxytocin. Unfortunately, the analogue was no longer available for our investigations, nor were other enzyme probes aiming at the post‐prolin cleaving enzyme at hand. Thus, a conceivable mode of *C*‐terminal splitting could not be clarified.

Remarkable is also the virtual identity of *κ*‐constants found for oxytocin and hydroxyoxytocin. A cleavage of substrates with the *N*‐terminal 2‐hydroxy‐3‐β‐mercaptopropionic acid by aminopeptidases has not been reported so far (and appears to be very unlikely), but another form of hydrolysis on the *N*‐terminal peptide bond cannot obviously be excluded. Instead, the polarity of the *N*‐terminal substituent of oxytocin analogues (N‐D grouping) seems to be essential for the rate of the enzymatic clearance.

### 4.3 Transport processes

The transport constant *k*_*r*_ is defined as a ratio of the diffusion constant *k* and the volume *V*_*r*_ of the receptor compartment. A low scatter of *k*_*r*_ (Table 2) is rather astonishing, for it seems likely that the assumed volume of the receptor compartment *V*_*r*_ (Equation 1) varies in measurements on various muscle strips. In this sense, *k*_*r*_ would rather stand for a parameter of an individual experimental run, and its scatter would represent the *V*_*r*_ distribution among individual myometrium preparations. Low scatter may be taken for an evidence that the relative volume of the receptor compartment in the rat uterus muscle is roughly constant, at least in a restricted group of individual tissue preparations. As can be inferred from the Free–Wilson analysis (Figure 5, right‐hand panel), the effect of the *N*‐terminal substitution on *k*_*r*_ is, in contrast to *κ*, less evident. A minor—insignificant—difference of the median values between the N and D groups (Figure 3, box plot in the upper panel) may possibly be accounted for differences in hydrophobicity of peptides with differing polarity of *N*‐terminal substituents.

## 5 CONCLUSIONS

Conclusions derived from the investigations presented here are as follows:

1. An adapted version of the oil‐immersion technique enables an assessment of clearance and transport rate constants in the receptor compartment of drugs eliciting tonic smooth muscle contraction. In the ex vivo rat myometrium, this state is optimally achieved in the K^+^‐depolarized state.
2. A comparison of ex vivo and in vivo estimated clearance rate constants of oxytocin‐like peptides supports the hypothesis that the response duration is mainly controlled by irreversible clearance in the receptor compartment. A peripheral clearance appears less efficient to these aims.
3. Clearance descriptors of oxytocin and oxytocin‐like peptides estimated in vivo (“native state” in oestrogen dominated uterus) and ex vivo in the oil‐immersion setup (K^+^‐depolarized uterus) do not significantly differ, indicating that inactivation processes do not depend on the state of the uterus tissue.
4. Potassium depolarization decreases the sensitivity of oestrogen‐dominated myometrium towards oxytocin‐like peptides in average by a factor of 25.
5. Statistical and segment contribution analyses confirm the splitting of the *Ν*‐terminal peptide bond as the dominating clearance process in oxytocin‐like peptides, provided that the substituent on the *N*‐terminal C^α^ atom is a polar group (NH_2_ or OH). It covers 50% to 60% of the total irreversible clearance. Rate constants of individual peptides are linear combinations of the corresponding segment contributions.

## CONFLICT OF INTEREST

None.

## ENDNOTES

* The term in situ is used here for in vivo processes at the site responding to the biologically active agents, ex vivo for experiments in an isolated intact organ (myometrium strip), in vitro for disintegrated tissue or tissue homogenates.

† Review in https://en.wikipedia.org/wiki/Oxytocin_receptor (2 November 2020).

‡ IU: international units. Oxytocic activity of 0.5 mg of the USP Posterior‐pituitary Reference Standard (WHO International Standard of Oxytocin. USP: U.S. Pharmacopeia Convention); 1 mg of synthetic oxytocin corresponds to 490 IU (former estimates: 450 IU).

§ Recommended IUPAC‐IUB nomenclature: [1‐(3‐mercaptopropanoic acid), 4‐glutamic acid]oxytocin; frequently used: [Mpa^1^,Glu^4^]oxytocin.
